# Relationship between WCS120 Protein Family Accumulation and Frost Tolerance in Wheat Cultivars Grown under Different Temperature Treatments

**DOI:** 10.3390/plants10061114

**Published:** 2021-05-31

**Authors:** Pavel Vítámvás, Ilja Tom Prášil, Jan Vítámvás, Miroslav Klíma

**Affiliations:** 1 Department of Crop Genetics and Breeding, Crop Research Institute, Drnovská 507/73, 16106 Prague 6-Ruzyně, Czech Republic; prasil@vurv.cz (I.T.P.); jan.vitamvas@vurv.cz (J.V.); klima@vurv.cz (M.K.); 2 Department of Forest Ecology, Faculty of Forestry and Wood Sciences, Czech University of Life Sciences Prague, Kamycka 129, 16500 Prague-Suchdol, Czech Republic

**Keywords:** cold acclimation, LT50, dehydrins, *Triticum aestivum*, controlled condition

## Abstract

Frost tolerance (FT) is generally acquired after exposure of plants to low, but non-freezing temperatures, where it is associated with the accumulation of COR proteins. The aim of the study was to reveal the effect of different temperature treatments (25, 17, 9 and 4 °C) on accumulation of cold-regulated dehydrins, dry weight content, and the development of FT in five wheat cultivars of different frost-tolerances in detail. The levels of cold-regulated dehydrins, WCS120 proteins in wheat were determined by immunoblot analysis, probed with an anti-dehydrin antibody. The lower the growth temperature: the higher the level of frost tolerance, dry weight content, and dehydrin accumulation, in all cultivars. There was a significant correlation between the level of induced FT and the accumulation of WCS120 proteins in cultivars grown at lower temperatures (9 and 4 °C). Moreover, the highly frost-tolerant wheat cultivars (as opposed to the lower-tolerant) accumulated higher levels of WCS120 proteins at 17 °C, a temperature at which it was not possible to differentiate between them via a frost test. Here, we demonstrated the possibility to distinguish differently frost-tolerant cultivars grown at different temperatures by the accumulation of different members of WCS120 family.

## 1. Introduction

The ability of winter habit crops to survive winter is a major factor affecting the area of their cultivation. One of their main winter survival traits is the level of frost tolerance (FT). FT of crops is induced immediately after the plant’s exposure to a cold treatment (e.g., [1]). The level of FT depends on duration of cold acclimation and temperature of cold acclimation. Global climate change could lead to deacclimation of winter crops in winter due to warmer period which can result in serious frost damage by subsequent freezing temperatures. The evaluation of frost tolerance by LT50 (lethal temperature - the temperature at which 50% of the leaves were killed) is a time-consuming procedure; including weeks of cold-acclimation, days of exposure of the plants to the scale of freezing temperatures, and weeks of regeneration in order to obtain the LT50 (e.g., [2,3]). Therefore, alternative methods, in order to do faster pre-screening of higher frost tolerant plants, are required by plant breeders.

The promising markers of FT and winter survivals of crops belong to cold-regulated (*Cor*) genes whose expression levels are correlated with frost tolerance of the crops (for review see [4]). Among the *Cor* genes, *Lea II* (dehydrin) genes, *wcs120* (wheat cold specific) gene family from wheat encode WCS120 proteins [5]. WCS120 proteins are of the K_n_-type and are located on the long arm of homoeologous group 6 chromosomes in wheat and they range in molecular weight (MW) from 12 to 200 kDa; among these, the five major members, WCS200 (MW 200 kDa), WCS180 (180 kDa), WCS66 (66 kDa), WCS120 (50 kDa), and WCS40 (40 kDa), are accumulated under cold treatment [6,7,8]. Our previous studies showed that with the increased amount of loaded sample cultivated under higher temperature (17 or 20 °C) the different level of WCS120 protein in these samples could distinguish winter wheats with different winter survival or maximal level of LT50 [9,10]. In non-vernalized plants, the total amount of detected dehydrins was correlated with winter survival of wheat cultivars sampled from field [11].

Fowler [12] studied the dynamics of cold acclimation in differently frost tolerant rye, wheat and barley cultivars and found out that highly frost tolerant genotypes reveal higher threshold induction temperatures for induction of enhanced acquired frost tolerance determined as LT50 when compared to the less tolerant ones. Vágújfalvi et al. [13] reported *Cor14b* transcript accumulation at relatively high temperatures 18/13 °C (day/night) only in frost tolerant winter wheat Cheyenne and substitution line harboring chromosome 5A from Cheyenne while in frost sensitive Chinese Spring, *Cor14b* transcript was detected only under cold (2 °C). Similarly, in the study by Vágújfalvi et al. [14], *Cor14b* transcript was clearly detected in relatively tolerant facultative einkorn wheat (*T. monococcum*) genotype G3116 from 5 °C up to 20 °C growth temperatures while in susceptible spring genotype DV92, *Cor14b* transcript was detected only in plants grown at cold temperatures (5, 10 °C). However, the comparison of the five major cold-inducible dehydrins accumulated in the same number of samples of the differently frost tolerant cultivars with actual and maximal level of LT50 was not reported up to now. The possibility to select cultivars with different FT grown under different temperature (i.e., with different level of cold acclimation) in the same number of samples could accelerate breeding of winter cereals sampled also in the field by high-throughput analysis such as ELISA (enzyme-linked immunosorbent assay) or targeted proteomics.

Therefore, our aim was to study a relationship between the accumulation of five major WCS120 proteins (WCS200, WCS180, WCS66, WCS120 and WCS40) and the acquired level of FT of the cultivars grown at different temperatures. To get more information on the development of plant responses to different temperature (cold) treatments, dry weight content (DWC) was also assessed indicating the dry-weight accumulation in mature leaves [7].

## 2. Results

There were no significant differences in tested parameters between the experiments conducted in 2007 when compared to those conducted in 2020 as shown in [15] (pp. 94–110) and presented results here.

**Figure 1:**
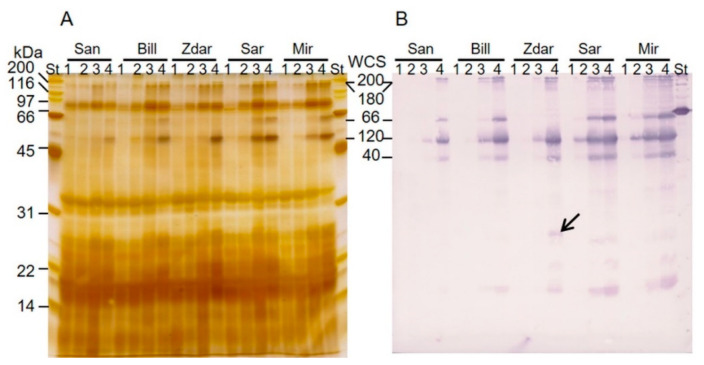
Accumulation of WCS120 proteins in the spring cultivar (Sandra) and the winter wheat cultivars cultivated at 25 °C (1), 17 °C (2), 9 °C (3) and 4 °C (4). (**A**) Silver-stained 1D SDS-PAGE gel. (**B**) Immunoblot with WCS120 proteins detected by anti-dehydrin primary antibody (against the K-segment). An arrow indicates a band visible only in Zdar (MW about 26 kDa). Mir-cultivar Mironovskaya 808, San-cultivar Sandra, Sar-cultivar Šárka, St-SDS-PAGE Molecular Weight Standards, broad range (Bio-Rad, Hercules, CA, USA).

The accumulation of dehydrins in the leaves of wheat cultivars Sandra (San), Bill, Zdar, Šárka (Sar) and Mironovskaya 808 (Mir) was detected by immunoblot analysis of the proteins soluble upon boiling (Figure 1). The polyclonal anti-dehydrin antibody bound to the most abundant members of WCS120 proteins, which were identified according to their molecular weights as WCS200 (200 kDa), WCS180 (180 kDa), WCS66 (66 kDa), WCS120 (50 kDa) and WCS40 (40 kDa; Figure 1B). Furthermore, the WCS120 proteins were also noticeable on silver-stained 1-DE gels (Figure 1A).

In all of the wheat samples with detected WCS120 proteins, WCS120 reached the highest relative accumulation (in all experiments and in 4 °C, it was 3.28% and 5.32% of total density detected, respectively), WCS66 was the second one (1.20% and 2.14%, respectively; with the exception of Zdar), WCS40 reached the third highest level of relative accumulation (1.14% and 1.92%, respectively), WCS200 reached the fourth highest relative accumulation (0.62% and 1.13%, respectively) and WCS180 had the lowest relative accumulation (0.39% and 0.71%). WCS66 was not present in any immunoblots of Zdar, and, therefore, Zdar had reduced values of the sum of all WCS120 proteins. However, an additional band of about 26 kDa present exclusively in the leaves of Zdar grown at 4 °C was recognized by anti-dehydrin antibody, (Figure 1B; relative accumulation 0.88% in Zdar only and, when expressed for all cultivars grown in 4 °C, the average accumulation reached only 0.04%).

**Figure 2:**
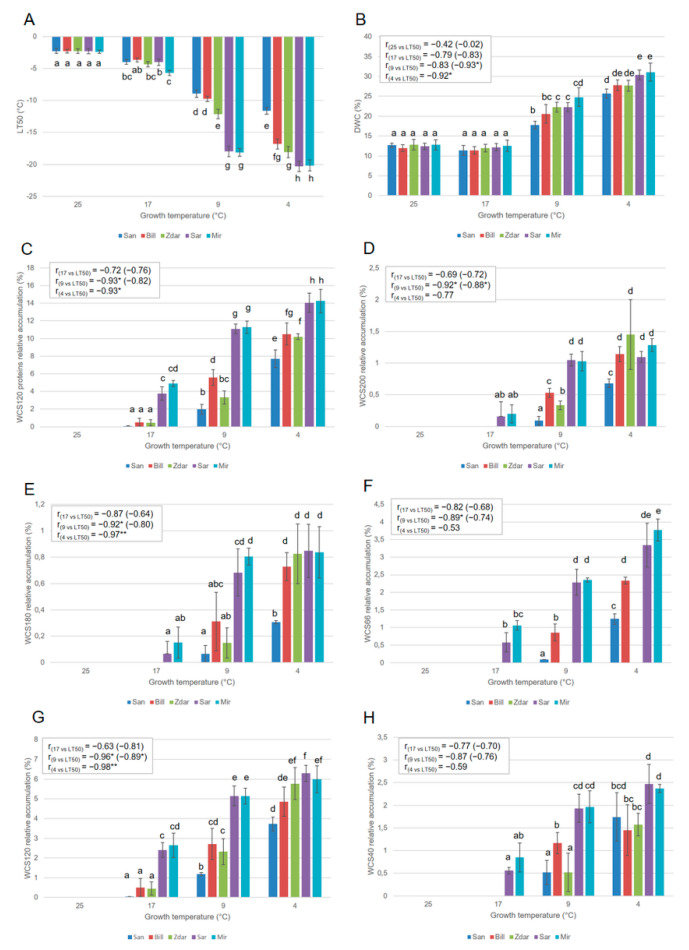
The values of the (**A**) LT50, (**B**) dry weight content (DWC), (**C**) sum of the WCS120 proteins (WCS120 proteins), (**D**) WCS200, (**E**) WCS180, (**F**) WCS66, (**G**) WCS120 and (**H**) WCS40 in the wheats cultivated under different growth temperature (25, 17, 9, and 4 °C). Presented data are mean values calculated from 6 repetitions. Error bars indicate SD, different letters indicate significant differences at 0.05 level using ANOVA analysis followed by Duncan’s multiple range test. One asterisk (*) and two asterisks (**) indicate statistical significance of correlation coefficient at *p* = 0.05 and 0.01, respectively. “20, 17, 9, 4 vs. LT50”—value for correlation between value of sample cultivated under 25, 17, 9, and 4 °C treatment and LT50 revealed in the treatment. Bracket in the linear correlation coefficient line indicates the correlation between the sum of WCS120 proteins and maximal LT50 (i.e., from 4 °C treatment). r—Pearson correlation coefficient.

A significant correlation between growth temperature, levels of FT, DWC and WCS120 proteins’ accumulation was observed (Figure 2).

The differences in the accumulation of the sum of all WCS120 proteins (dehydrin, Figure 2C), extracted from plants grown at different temperatures, showed almost the same trend as the single major WCS120 proteins (Figure 2). In all of the cultivars grown at 25 °C, the WCS120 proteins were not detected, and FT and DWC showed the lowest values with no significant differences between the cultivars.

When the wheats were grown at 17 °C, the highly frost-tolerant cultivars Mir and Sar accumulated WCS120 proteins, contrary to the undetectable levels of WCS120 proteins in the low frost-tolerant Zdar and Bill, as well as frost-sensitive San. Correspondingly, the value of LT50 was slightly lower in the Mir cultivar than in the others. However, the LT50 of the leaves of the other cultivars had a similar value, and showed a slight increase in FT in plants grown at 17 °C contrary to those plants grown at 25 °C. No significant changes in the levels of DWC were observed in the plants cultivated at 25 and 17 °C. No significant correlations between LT50 and all of the dehydrins investigated were found in plants grown at 17 °C. However, the cultivars with the highest FT (Mir, Sar) revealed significant changes in all five major dehydrins and in the sum of all WCS120 proteins accumulation contrary to other cultivars with lower FT (Figure 2).

All cultivars grown at 9 °C showed significantly higher levels of five major members of WCS120 proteins, sum of WCS120 proteins, FT and DWC, than did those plants cultivated at 17 °C. The highest FT (lowest LT50), DWC and accumulation of all WCS120 proteins were reached in the plants cultivated at 4 °C. The differences in DWC and FT between cultivars increased with temperature decline. On the other hand, a similar increase (the same differences) in the level of WCS120 proteins in all wheat cultivars were observed at 4 °C, as well as at 9 °C (Figure 2). Statistically significant linear correlation coefficients (r = −0.96, −0.98) between LT50 and WCS120 were found in plants grown at 9 °C and 4 °C, respectively. However, a significant linear correlation (r = −0.93, −0.92, −0.92, −0.89) was also found for the sum of all WCS120 proteins, WCS200, WCS180 and WCS66 extracted from plants cultivated at 9 °C, respectively. In samples cultivated at 4 °C, a significant correlation (r = −0.93, −0.97) was also found for the sum of all WCS120 proteins and WCS180, respectively (Figure 2).

It was possible to detect the differences between the cultivars according to the level of WCS120 protein or the sum of all WCS120 proteins in the plants grown at 17 °C; while according to actual LT50, the differences can be determined only in the plants grown at 4 and 9 °C. Nevertheless, at 17 °C, two groups of cultivars were distinguished: highly frost tolerant Mir and Sar; and medium or low tolerant Zdar and Bill, including sensitive San, as well. At 9 °C and 4 °C three groups of cultivars could be distinguished: highly frost tolerant Mir and Sar; medium or low tolerant Zdar and Bill; and the least tolerant San (Figure 2). These results indicate that WCS120 proteins started to accumulate early, and at higher temperatures, in highly frost-tolerant wheat cultivars (even before any significant decrease of LT50). On the other hand, sensitive San accumulated WCS120 proteins at a much lower temperature, and at significantly lower levels when compared with the other wheat cultivars.

## 3. Discussion

The low variability of obtained results between plants developed from 2005 and 2019 harvested seeds confirmed importance of studying older plants such as three-leaf stage plants in this experiment that helped us to minimize the influence of possible differences in seed storage compounds accumulation among the different growth seasons. The preliminary data conducted in 2006 [15], (pp. 94–110) are consistent with the data obtained in 2020. Therefore, the dehydrin analysis seems to be a reliable tool in the studies of wheat frost-tolerance despite different seeds harvest season.

In our experiments, the temperature-dependent changes in the level of FT, DWC, and the accumulation of dehydrins were compared in five wheat cultivars. Of the dehydrins, we focused on those proteins which are cold-regulated and abundant in cereals, i.e., the WCS120 protein family in wheat [6]. Cereals should usually be exposed to temperatures below 12 °C (the lower, the better, i.e., they have to be hardened by cold) to evaluate their FT levels by frost tests [16,17].

The increase of DWC in cold-acclimated plants reflects an accumulation of cold-induced metabolites and proteins that improve frost tolerance and winter survival (e.g., [3,4,7]). The revealed significant correlation between DWC and LT50 under 4 °C treatment (Figure 2B) could indicate a (relatively) cheap and efficient tool for plant FT evaluation. However, the low significant difference in DWC values in the lowest growth temperature (only two groups were distinguished by DWC at 4 °C) contrary to significant differences in dehydrins values (three groups were distinguished by dehydrins at 4 °C) indicates that DWC values could be reliable only as a cold-acclimation indicator. The fact that DWC responds faster on different environmental factors (temperature, stress) than dehydrin accumulation is clear also from our previous study [3]. The significant correlation between DWC and LT50 was revealed probably only due to fast sampling of only five cultivars that decreased possible variability of DWC.

We were able to distinguish and quantify all five of the most abundant members of the WCS120 protein family in wheat utilizing immunoblot analysis, using anti-dehydrin primary antibody. Among the WCS120 proteins, the most abundant was WCS120, followed by WCS66; which corresponds to published data e.g., [3,7,18]. All WCS120 proteins rapidly accumulated in leaves under cultivation at lower temperatures (9 or 4 °C). The sum of all WCS120 proteins had almost the same dynamics as the WCS120 protein alone. The fact that the frost tolerant cultivar Zdar did not accumulate any WCS66 demonstrates that the number of dehydrin proteins accumulated upon cold is not fixed in wheat cultivars. The identification of the 26 kDa protein (found only in the Zdar) was not successful by MALDI-TOF/TOF approach due to low concentration of the dehydrin in the 26 kDa protein band (compare Figure 1A,B). Therefore, the LC-MS/MS identification of the proteins separated on two-dimensional polyacrylamide electrophoresis is currently in progress. Thus, we can only speculate that this protein might be the product of a mutation in the *wcs66* gene. The correlation between LT50 and WCS120 proteins showed increases in the level of both WCS120 proteins and FT, in the leaf tissue of all cultivars, with decreasing cultivation temperatures.

From the immunoblot analysis (Figure 1), it is clearly visible that the differentially frost tolerant wheat cultivars showed different threshold temperatures for the induction and detection of the WCS120 proteins. Moreover, the wheats were already differentiated at a higher temperature (17 °C). The result is in accordance with our previous results [9,10]. However, the highly frost tolerant cultivars Mir and Sar detected in those treatments were loaded in the same amount as cold acclimated samples contrary to higher load in both previous studies with wheat dehydrin detection also in sensitive cultivars grown under 17 or 20 °C. Almost all major members of WCS120 protein family revealed a significant correlation with LT50 in cold-acclimated samples. WCS120 protein showed the best correlation in both cold treatments (9 and 4 °C). However, the sum of all WCS120 proteins revealed also a significant correlation and a significant difference in density between genotypes (Figure 2) with the cultivars´ differential winter survival [11]. The result could also explain the better correlation of the sum of WCS120 proteins accumulation contrary to WCS120 accumulation in the recent study on dehydrin accumulation in cereals sampled from the field [11]. Under the higher variability in dehydrin accumulation of the major members of WCS120 family due to different combination of environmental factors in the field, the higher significant difference in the sum of WCS120 proteins could result in stronger correlation as revealed in Vítámvás et al. [11]. Therefore, the possibility of the application of dehydrin accumulation in high-throughput screening for different cultivar FT could be accomplished by ELISA with anti-dehydrin primary antibody (i.e., detecting sum of WCS120 proteins) or targeted proteomics (e.g., detecting peptides with K-segments).

It can be concluded that at higher growth temperatures (17 °C), the differently frost-tolerant wheat cultivars can be better distinguished by dehydrin accumulation than by a direct frost test. All major members of WCS120 protein family except WCS40 revealed a significant correlation with LT50 in cold-acclimated samples. Based upon our results, a relative accumulation level of the sum of all WCS120 proteins and/or WCS120 protein alone could be used for a pre-screening program for frost tolerant wheat cultivars.

## 4. Materials and Methods

### 4.1. Plant Material

These experiments were conducted with wheat (*Triticum aestivum* L.) cultivars Mironovskaya 808 (Mir), Šárka (Sar), Zdar, Bill and Sandra (San). These cultivars exhibit different winter hardiness, according to a long-term pot experiment, under natural conditions [19]. From the winter hardiness scale for wheat cultivars, 9 (indicating the highest winter hardiness) to 1 (the lowest winter hardiness) Mir, Sar, Zdar, Bill, and San showed degrees of winter hardiness of 8, 6, 4, 3 and 1, respectively. All seeds were obtained from the breeding company Selgen a.s., Prague, Czech Republic. Seeds obtained in 2006 and 2020 were used to reveal possible variability between different growing seasons.

### 4.2. Growth Conditions

The seeds were germinated at 20 °C for 3 d. Then, the seedlings were grown in a growth cabinet (Tyler, Jászberény, Hungary) with a 12 h photoperiod (irradiance of 400 µmol m^−2^ s^−1^ provided by a combination of vapor lamps and high intensity discharge lamps (LU/400/T/40, Tungsram, Jászberény, Hungary)); and at different temperatures (25, 17, 9 or 4 °C).

The seedlings of wheat cultivars Mir, Sar, Zdar, Bill and San were grown and developed their leaves (till three-leaf stage to minimize the seed-storage effect on energy-dependent cold-acclimation process) at one of the experimental temperatures (i.e., at 25 °C, 17 °C, 9 °C and 4 °C).

The 2nd leaf, i.e., the last fully developed leaf of the plants (developed from the seeds obtained in 2006 or 2020) was sampled in three biological and technical replicates for the analysis of the following characteristics: dry weight content, frost tolerance, and analysis of dehydrin content.

### 4.3. Dry Weight Content

The dry weight content (DWC = 100 × DW/FW) in the leaves was calculated as the ratio of dry weight (DW) to fresh weight (FW). DW was determined by lyophilization (−45 °C, overnight) of the leaves in a Laboratory Freeze Dryer CRYODOS-50 (Telstar Industrial, Terrassa, Spain).

### 4.4. Frost Tolerance

The level of FT in the cultivars was evaluated by a direct frost test [20]. Sets of six to eight leaf segments per tube, each segment of 1 cm length, were exposed in two repetitions to five different frost temperatures in five freezers for 8 h. Thereafter, small ice crystals were added to the tubes with segments at −2 °C, to initiate extracellular freezing of the segments for two hours. The rates of cooling and thawing were done at 2 °C h^−1^. After thawing, 14 mL of deionized water was added to each tube, and the degree of frost injury was evaluated by the conductivity method [20]. The lethal temperature LT50 (i.e., the temperature at which 50% of the leaves were killed) was calculated according to the model of Janáček and Prášil [2].

### 4.5. Protein Extraction

Proteins were extracted by Tris buffer (0.1 M Tris-HCl, pH 8.8, containing Complete^TM^, EDTA-free protease inhibitor cocktail (Roche, Mannheim, Germany) from frozen plant leaves, as described by Vítámvás and Prášil [3]. Dehydrins are stable upon boiling, and, therefore, a boiling step (15 min) was used to enrich the proteins in the sample. The protein concentration was determined according to Bradford [21].

### 4.6. Protein Analysis

Proteins were separated by SDS-PAGE on 10% resolving gels [22]. Samples were loaded into each well, equally, corresponding to 0.4 mg of FW of the leaves. Afterwards, the proteins were identified by an immunoblot analysis, using the polyclonal anti-dehydrin primary antibody (against the consensus K-segment [23]; Agrisera, Vännäs, Sweden). The proteins were electrophoretically transferred to nitrocellulose membrane (0.45 μm, Amersham Pharmacia Biotech, Piscataway, NJ, USA). After blocking with non-fat dry milk (3%) in TBS, the membrane was incubated with a 1:1000 dilution of anti-dehydrin primary antibody. After washing with TBS, containing 0.05% Tween-20 and 0.2% Triton X-100 (Sigma-Aldrich, Saint Louis, MO, USA), the secondary antibody (GAR-AP conjugate, Bio-Rad, Hercules, CA, USA) was applied at a dilution of 1:3000. The complex of proteins and antibodies was visualized by BCIP/NBT staining (Bio-Rad, Hercules, CA, USA). SDS-PAGE Standards, broad range (Bio-Rad, Hercules, CA, USA), were used for the estimation of molecular weight (MW). The amount of accumulated WCS120 proteins on the immunoblots was analyzed by *Quantity One v. 4.6.2* software (Bio-Rad, Hercules, CA, USA). In order to convert the densitometric values (reflective density/mm^2^) to percentage accumulation values, the total densitometric value in each image was set at 100%, and all other values were considered a percentage relative to the value.

### 4.7. Statistical Analysis

Three biological replicates and three technical replicates were used to analyses and the results were compared using ANOVA analysis, multiple comparisons and Duncan´s multiple range test at 0.05 level (*Statistica v. 10* software, *StatSoft,* Tulsa, OK, USA). Correlation analysis was carried out using Pearson’s correlation coefficient (R) and significance was considered at 0.05 level.

## Data Availability

Data is contained within the article.
